# Single-cell transcriptomic analysis reveals epithelial and microenvironmental heterogeneity in small cell carcinoma of the esophagus

**DOI:** 10.3389/fimmu.2025.1672587

**Published:** 2025-10-08

**Authors:** Xiaolei Yin, Xiaopeng Li, Lili Mi, Jiaojiao Hou, Fei Yin

**Affiliations:** ^1^ Department of Gastroenterology, The Fourth Hospital of Hebei Medical University, Shijiazhuang, Hebei, China; ^2^ Medical Record Room, The Fourth Hospital of Hebei Medical University, Shijiazhuang, Hebei, China

**Keywords:** small cell carcinoma of the esophagus, single-cell RNA sequencing, heterogeneity, cancer-associated fibroblasts, immune suppression

## Abstract

**Background:**

Small cell carcinoma of the esophagus (SCCE) is a rare and highly aggressive malignancy with limited therapeutic options and poor prognosis. The paucity of clinical specimens and lack of established experimental models have hindered a comprehensive understanding of its cellular heterogeneity and tumor microenvironment.

**Methods:**

We performed single-cell RNA sequencing on SCCE samples, and integrated them with publicly available scRNA-seq datasets from esophageal squamous cell carcinoma (ESCC), esophageal adenocarcinoma (EAC), and adjacent normal tissues (NT) from ESCC and EAC cases. An integrative transcriptomic analysis was conducted to identify cell types, infer malignant states, reconstruct differentiation trajectories, evaluate immune landscapes, and investigate fibroblast subtypes and cell–cell communication networks.

**Results:**

SCCE tumors were characterized by a predominance of malignant epithelial cells and exhibited a profoundly immunosuppressed phenotype, with reduced immune infiltration and widespread downregulation of immune checkpoint genes. Malignant epithelial cells showed pronounced chromosomal instability and were classified into three transcriptionally distinct subtypes with divergent differentiation trajectories. The tumor microenvironment featured a complex stromal compartment, with enrichment of extracellular matrix fibroblasts (eCAFs) characterized by elevated ELF3 regulatory activity, and collagen-driven signaling predominantly mediated by inflammatory CAFs (iCAFs). SCCE also showed the most intricate cell–cell communication network among esophageal cancer subtypes.

**Conclusion:**

Our single-cell atlas offers a detailed view of the cellular heterogeneity and microenvironmental complexity of SCCE, highlighting its distinct tumor architecture, immune exclusion, and stromal reprogramming. These findings provide a valuable resource for understanding SCCE biology and form a basis for future mechanistic and exploratory biological investigations.

## Introduction

Small cell carcinoma of the esophagus (SCCE) is a rare and highly aggressive neuroendocrine malignancy, accounting for less than 3% of all esophageal cancers (1, 2). It is marked by rapid progression, early metastasis, and poor clinical outcomes, with most patients presenting at an advanced stage and a median survival of only 8 to 13 months (3). Due to its rarity, SCCE currently lacks established treatment guidelines, and clinical management often relies on treatment approaches developed for small cell lung cancer (SCLC). However, small cell carcinomas arising from different tissues exhibit distinct biological characteristics, and treatment responses may not be directly transferable across cancer types (4). The scarcity of fresh tumor samples, the absence of established experimental models, and limited genomic data have collectively impeded a deeper understanding of SCCE pathogenesis and potential therapeutic vulnerabilities.

While recent advances in single-cell technologies have illuminated cellular heterogeneity and microenvironmental complexity across various solid tumors (5, 6), SCCE remains poorly characterized at this resolution. Prior studies using bulk transcriptomic or genomic profiling have identified recurrent mutations and limited immune infiltration in SCCE (7, 8), but lack the granularity to resolve intratumoral heterogeneity or cell-type-specific alterations. A recent single-cell study has provided valuable insights into the SCCE ecosystem (9); however, further analyses are needed to refine our understanding of its epithelial diversity, stromal heterogeneity, and immune landscape. Notably, SCCE may exhibit distinct cellular programs and microenvironmental characteristics compared to SCLC, despite sharing the same histological classification. In addition to these differences, the cellular origin of SCCE remains incompletely understood. While SCCE exhibits neuroendocrine differentiation similar to SCLC, it is unclear whether these tumors arise from a distinct neuroendocrine lineage within the esophageal epithelium or through transdifferentiation from other esophageal cell types. Emerging evidence suggests that neuroendocrine features in epithelial cancers, including those of the gastrointestinal tract, may arise through lineage plasticity mechanisms such as transcriptional reprogramming and transdifferentiation (10). However, direct evidence regarding the ontogeny of SCCE is lacking due to the scarcity of relevant models and longitudinal tissue data. Single-cell transcriptomic profiling offers a valuable approach to explore such lineage relationships and may offer insights into the developmental trajectories and differentiation programs of SCCE. Therefore, high-resolution characterization of SCCE is essential to elucidate its cellular origin, define its epithelial architecture, and dissect stromal–immune features, all of which may offer foundational insights for future therapeutic development.

To address this need, we performed single-cell RNA sequencing on SCCE tumor tissues to generate a high-resolution cellular atlas of this rare malignancy. Through integrative analysis of tumor, stromal, and immune compartments, we identified the molecular subtypes of malignant epithelial cells, mapped the composition and functional states of tumor-infiltrating immune cells, and characterized distinct cancer-associated fibroblast (CAF) subtypes along with their associated signaling activities. In particular, we uncovered an immunosuppressive microenvironment and a complex fibroblast-driven signaling network distinctive to SCCE. These findings provide a framework for understanding the cellular and molecular features that define the unique biology of SCCE.

## Materials and methods

### Sample collection and dataset composition

Single-cell RNA sequencing data were obtained from a total of 23 esophageal tissue samples, encompassing three histological subtypes: EAC, ESCC, and SCCE, as well as NT derived from ESCC- and EAC-associated samples. Among them, three SCCE tumor samples were newly generated in-house using single-nucleus RNA sequencing (snRNA-seq) from formalin-fixed paraffin-embedded (FFPE) tissue blocks sourced from The Fourth Hospital of Hebei Medical University. For each FFPE specimen, 25-μm tissue curls were collected into a tube before serial sectioning for the Chromium Single Cell Gene Expression Flex (scFFPE-seq) workflow (10x Genomics). Three such curls (75 μm total) were pooled and processed as a single replicate.

The remaining 20 samples were derived from publicly available single-cell datasets. Seven ESCC tumors and their paired NT samples were obtained from the GSE145370 dataset (11), while four EAC tumors and two NT samples were retrieved from the GSE222078 dataset (12). All public datasets were preprocessed and deemed suitable for downstream analyses.

### Library preparation and sequencing

For each SCCE sample, three 25-μm FFPE curls (75 μm total) were dissociated using the Bioyou^®^ Nuclei Isolation Kit for FFPE Tissue (Shanghai Biotechnology Corporation). Approximately 600,000 nuclei were isolated, washed, and counted. Libraries were constructed according to the Chromium Single Cell Gene Expression Flex User Guide (10x Genomics, CG000477). Sequencing was performed on an Illumina NovaSeq 6000 platform using paired-end 150 bp reads (2×150 bp).

### Single-nucleus RNA-seq data processing, clustering, and annotation

Raw sequencing data (FASTQ files) were processed using the Cell Ranger multi-pipeline (v7.1.0, 10x Genomics) with the Human Transcriptome Probe Set reference. Gene-barcode matrices were generated for each sample by UMI counting and background barcode filtering. The resulting expression matrices were imported into the Seurat package (v5.1.0) in R (v4.4.1) for quality control and downstream analysis (13). Cells with fewer than 200 or more than 6,000 detected genes were excluded. Additionally, cells with a mitochondrial gene content of more than 10% were filtered out, determined by the PercentageFeatureSet function. The NormalizeData function was used to normalize gene expression data, and highly variable genes were pinpointed while accounting for the mean–variance relationship. Data integration across samples was performed using FindIntegrationAnchors and IntegrateData. UMAP plots before and after integration are presented in **Supplementary Figure 1** to visualize batch correction performance. The integrated data were scaled and subjected to principal component analysis (PCA), with the top 30 principal components retained. A shared nearest neighbor (SNN) graph was then constructed, followed by graph-based clustering using the Louvain algorithm. Clustering resolution was systematically optimized between 0.1 and 1.0, and a resolution of 0.4 was selected for downstream analysis. The resulting clusters were visualized using Uniform Manifold Approximation and Projection (UMAP) (14).

For subpopulation analyses, clustering was repeated on selected subsets of cells after rescaling and dimensionality reduction. Cluster-specific differentially expressed genes (DEGs) were identified using the FindAllMarkers function in Seurat with parameters set to logfc.threshold = 0.25, min.pct = 0.25, and only.pos = TRUE. An adjusted p-value below 0.05 indicated that the genes were statistically significant. Initial cell type annotation was performed using the SingleR package (15), followed by manual refinement based on the expression of canonical marker genes and reference to established literature and previously published single-cell datasets.

### CNV-based identification of malignant epithelial cells

Large-scale chromosomal copy number variation (CNV) was estimated using the inferCNV package (v1.20.0) (16). Epithelial cells from the NT group were selected as the reference population. The CNV score for each epithelial cell was computed by comparing its gene expression pattern across chromosomal positions with that of the reference group. To further refine the classification, the top 5% of epithelial cells with the highest CNV scores within each pathological group were extracted, and their average expression profiles were used to compute Pearson correlation coefficients between each remaining epithelial cell and this high-CNV subset. Epithelial cells were ultimately classified as malignant if both of the following criteria were met: a CNV score greater than 0.001 and a correlation coefficient with the high-CNV group greater than 0.5. Cells not meeting both thresholds were considered non-malignant. This dual-criterion strategy enabled a robust delineation of malignant epithelial populations based on both chromosomal aberration patterns and transcriptional similarity.

### Pathway enrichment and functional scoring

Pathway analysis was performed using Kyoto Encyclopedia of Genes and Genomes (KEGG) enrichment and Gene Set Variation Analysis (GSVA) (17). KEGG analysis was carried out with the clusterProfiler package (v4.12.6) (18). GSVA was implemented using the GSVA package (v1.52.3), with Hallmark gene sets obtained from the Molecular Signatures Database (MSigDB v7.5.1) (19). Module scores were computed using the AddModuleScore function in Seurat. Gene sets related to epithelial–mesenchymal transition (EMT), angiogenesis, antigen presentation, interferon response, and inflammation were sourced from MSigDB. At the same time, additional modules such as Macrophage_M1 and Macrophage_M2 were curated from previously published studies (20).

### Differentiation scoring and pseudotime trajectory analysis

To evaluate the differentiation potential of malignant epithelial subpopulations in SCCE, the CytoTRACE2 (v1.0.0) package was applied to epithelial cells from the SCCE group (21). Differentiation scores were calculated and compared across malignant subclusters to assess their relative developmental states. The Monocle2 (v2.32.0) package was used to construct pseudotime trajectories based on highly variable genes (22). Dimensionality reduction was performed using the DDRTree method, and cells were ordered along a developmental continuum with non-malignant epithelial cells designated as the biological root. This approach enabled the inference of transcriptional progression among malignant epithelial subpopulations in SCCE.

### Transcription factor regulatory network analysis

Transcription factor regulatory network analysis was conducted using the pySCENIC pipeline (v0.12.1) (23), following the standard workflow previously described. Gene regulatory networks were initially inferred using GRNBoost2, which identified candidate transcription factor–target gene co-expression modules. These modules were then refined through cisTarget motif enrichment analysis to define high-confidence regulons. AUCell was used to calculate both regulon activity scores (RAS) for individual cells and regulon specificity scores (RSS) across cell groups. All steps were executed using default settings unless otherwise specified. The resulting matrices were imported into R and visualized with the SCENIC package (v1.3.1) (24).

### Cell–cell communication analysis

Cell–cell communication networks were inferred using the CellChat R package (v1.6.1). Normalized gene expression matrices and predefined cell type annotations were used as input. The analysis focused on intercellular communication between malignant epithelial cells and other major cell populations. The standard CellChat workflow was followed, including the identification of overexpressed genes, prediction of biologically significant ligand–receptor interactions, and computation of intercellular communication probabilities. The built-in human ligand–receptor database in CellChat was used for signaling inference. Group-specific analyses were conducted to evaluate differences in communication patterns across histological subtypes. All analyses were performed using default parameters unless otherwise specified. Visualization of inferred signaling networks was carried out using the built-in visualization functions in CellChat (25).

### Statistical analysis

All statistical analyses were conducted with R (v4.4.1). Non-parametric tests were used throughout the study. Group comparisons were conducted using the Wilcoxon rank-sum test or the Kruskal–Wallis test, as appropriate. Spearman’s rank correlation coefficient was applied to evaluate associations between continuous variables, including CNV scores, differentiation scores, and gene expression levels. Unless otherwise specified, a two-sided p-value of less than 0.05 was used to define statistical significance.

## Results

### Single-cell transcriptomic landscape of esophageal tissues across histological subtypes

**Figure 1:**
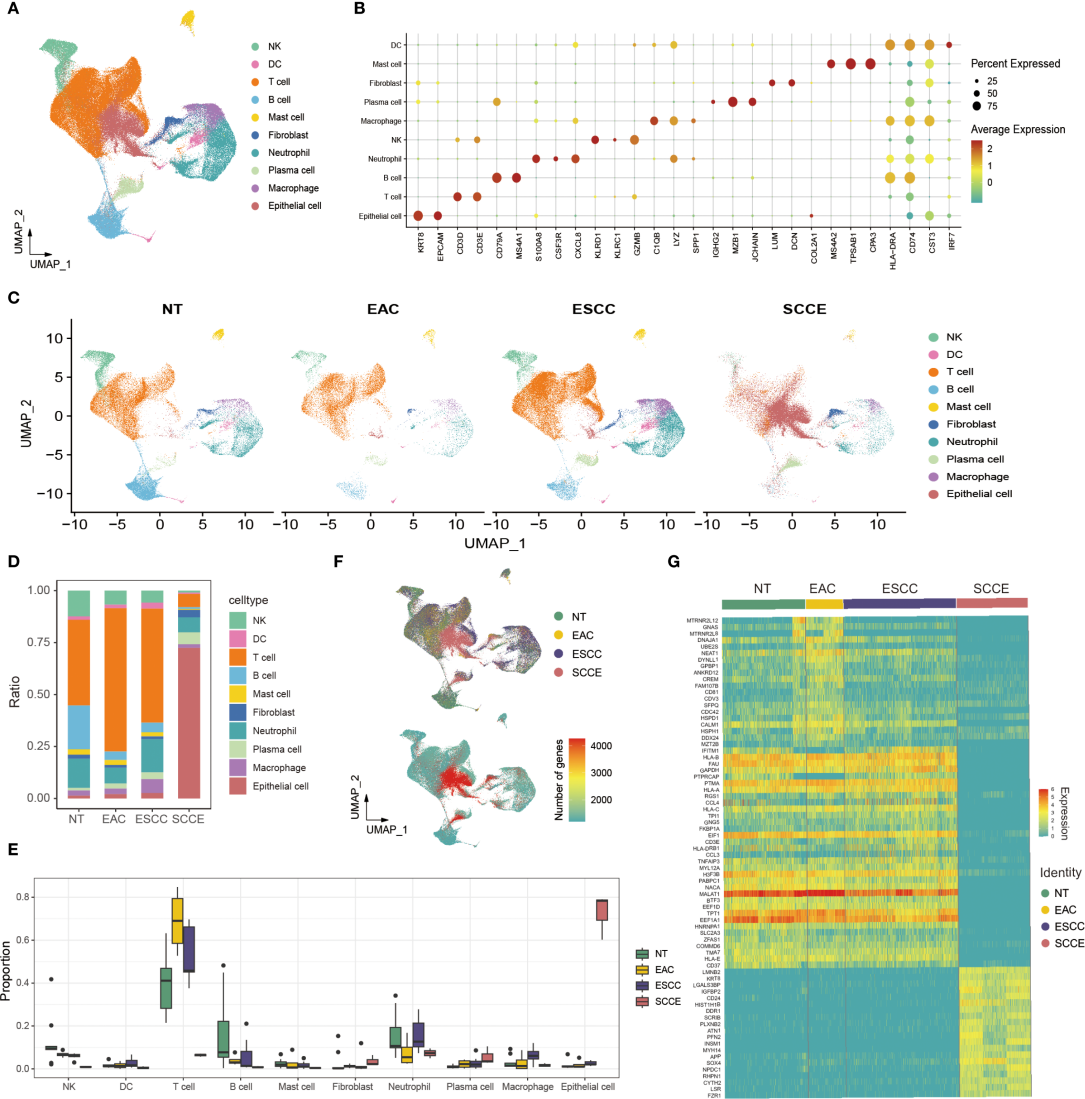
Single-cell transcriptomic landscape of esophageal tissues across histological subtypes. **(A)** UMAP plot showing the clustering of all cells into ten major cell types. **(B)** Dot plot illustrating canonical marker gene expression across identified cell types. **(C)** UMAP projections of cells from each histological subtype, highlighting differences in cellular composition. **(D)** Stacked bar plots showing the proportion of each cell type within individual samples. **(E)** Box plots comparing the relative abundance of selected cell types across histological groups. **(F)** Left: UMAP plot indicating cell origin by histological subtype. Right: UMAP plot colored by the number of DEGs identified in each cell type across groups. **(G)** Heatmap displaying the top 20 differentially expressed genes for each histological group across all cell types.

To characterize the cellular heterogeneity of esophageal tissues, we performed an integrative single-cell RNA sequencing analysis across from four histological types: NT, EAC, ESCC, and SCCE. Following standard preprocessing, dimensionality reduction, and clustering, we identified ten major cell populations, including epithelial cells, fibroblasts, mast cells, plasma cells, neutrophils, macrophages, dendritic cells (DCs), T cells, B cells, and NK cells (**Figure 1A**). Cell type annotation was guided by canonical marker gene expression patterns (**Figure 1B**).

UMAP projections of each histological subtype revealed distinct cellular distributions, with SCCE samples showing a marked enrichment of epithelial cells and a corresponding depletion of immune populations compared to other subtypes (**Figure 1C**). In line with these observations, compositional analysis revealed a significantly higher proportion of epithelial cells in SCCE. In contrast, immune subsets, such as T cells and macrophages, were more prevalent in NT and ESCC samples (**Figure 1D**). Quantitative comparisons further confirmed significant differences in cell-type composition across histological groups, particularly within epithelial and T cell compartments (**Figure 1E**).

Further analysis of transcriptional alterations revealed that SCCE samples exhibited a distinct transcriptomic profile. The distribution of differentially expressed genes (DEGs) across cell types was visualized in UMAP space, highlighting widespread gene expression remodeling in SCCE (**Figure 1F**, right). A heatmap of the top 20 DEGs per group demonstrated subtype-specific gene signatures, with SCCE displaying a unique pattern of upregulated and downregulated genes compared to other histological subtypes (**Figure 1G**). Collectively, these findings delineate the cellular complexity and transcriptional heterogeneity across esophageal cancer subtypes, with SCCE exhibiting a particularly distinctive molecular and cellular profile. Representative hematoxylin and eosin (H&E) staining images of EAC, ESCC, and SCCE tissues further illustrate the histological distinctions among the three cancer subtypes (**Supplementary Figure 2**).

### Malignant epithelial cell identification and functional characterization

**Figure 2:**
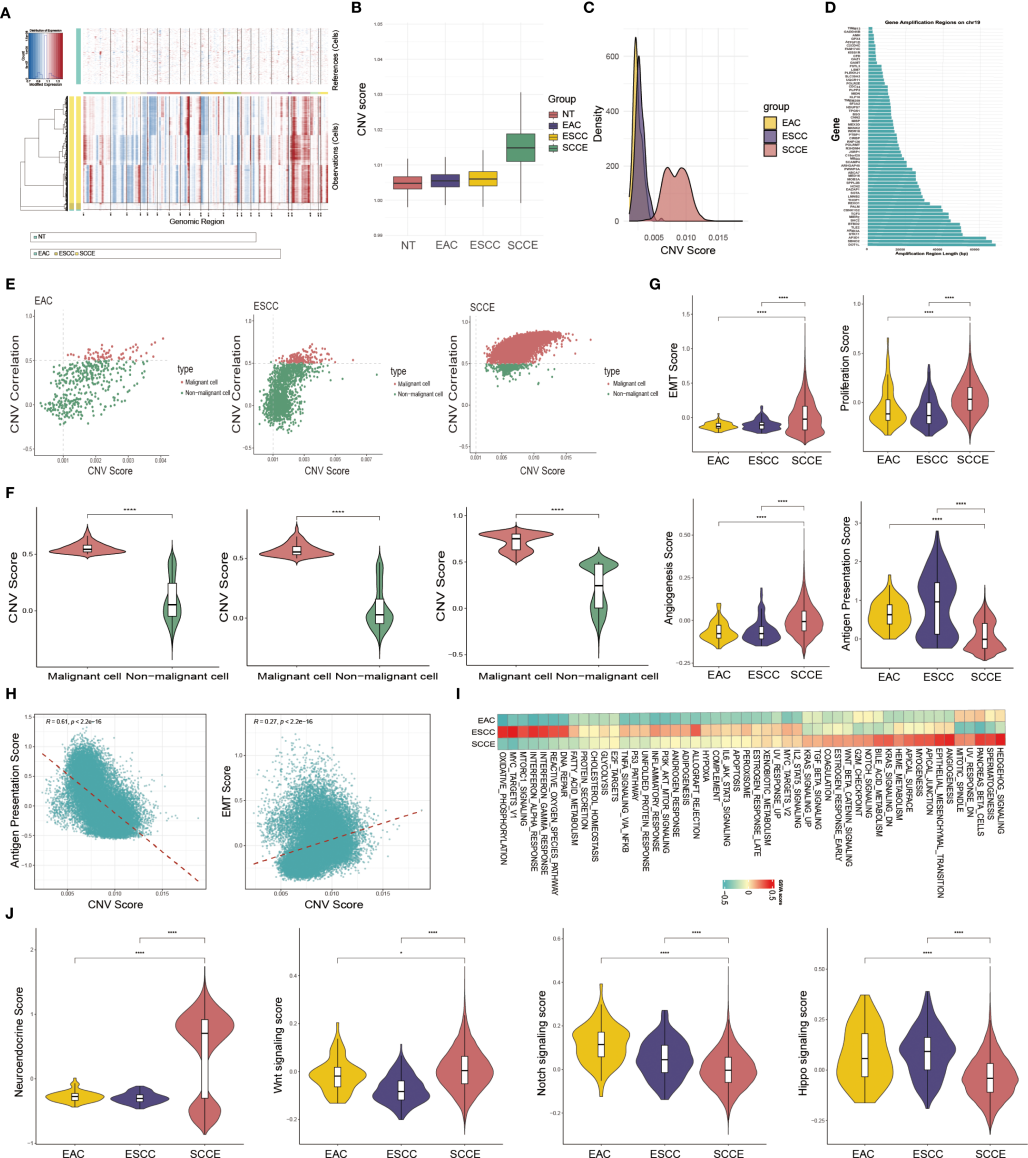
Identification and functional characterization of malignant epithelial cells across esophageal cancer subtypes. **(A)** Heatmap showing inferred CNV profiles of epithelial cells based on NT as the reference. **(B)** Box plot displaying CNV scores of epithelial cells across EAC, ESCC, and SCCE samples. **(C)** Density plot illustrating the distribution of CNV scores in each subtype. **(D)** Genomic view of inferred CNV signals, highlighting amplified genes along chromosome 19. **(E)** Scatter plots of CNV scores versus correlation values, used to distinguish malignant and non-malignant epithelial cells based on defined thresholds. **(F)** Violin plots comparing CNV scores between malignant and non-malignant epithelial cells within each subtype. **(G)** Violin plots showing EMT, proliferation, angiogenesis, and antigen presentation scores of malignant cells across the three subtypes. **(H)** Spearman correlation analysis between CNV scores and functional phenotypes in malignant epithelial cells from SCCE. **(I)** Heatmap of GSVA pathway enrichment scores in malignant epithelial cells across EAC, ESCC, and SCCE. **(J)** Violin plots comparing neuroendocrine (NE) signature scores and the activity of Wnt, Notch, and Hippo signaling pathways in malignant epithelial cells across EAC, ESCC, and SCCE. *Statistical comparisons in **(F, G)** were performed using the Wilcoxon rank-sum test. ****p < 0.0001.

To investigate epithelial cell heterogeneity across esophageal cancer subtypes, we performed inferCNV analysis using epithelial cells from NT as the reference. The resulting heatmap revealed pronounced CNVs in SCCE, characterized by widespread chromosomal amplifications and deletions, in contrast to the more modest alterations observed in EAC and ESCC (**Figure 2A**). SCCE epithelial cells exhibited significantly higher CNV scores than those from EAC and ESCC, as shown in the boxplot (**Figure 2B**). Notably, the density plot revealed a broader and bimodal distribution of CNV scores in SCCE, indicative of greater intratumoral heterogeneity in chromosomal alterations. In contrast, EAC and ESCC displayed relatively narrow, unimodal patterns (**Figure 2C**). Further analysis of the most frequently amplified genomic region in SCCE identified chromosome 19 as the predominant site, with extensive amplification signals spanning multiple genes (**Figure 2D**).

Scatter plots of CNV scores versus correlation coefficients revealed a clear separation between malignant and non-malignant epithelial cells. Cells exhibiting both high CNV burden and strong correlation with the high-CNV reference subset were classified as malignant, a pattern consistently observed across EAC, ESCC, and SCCE samples (**Figure 2E**). Violin plots further confirmed that malignant cells exhibited significantly higher CNV scores than non-malignant cells within each histological subtype (**Figure 2F**).

We next assessed functional phenotypes of malignant epithelial cells by comparing key biological pathway scores across EAC, ESCC, and SCCE. SCCE cells demonstrated significantly higher scores for epithelial–mesenchymal transition (EMT), proliferation, and angiogenesis compared to the other subtypes (all P < 0.0001), consistent with a more aggressive and metastatic phenotype. In contrast, antigen presentation scores were markedly reduced in SCCE, suggesting impaired antigen-presenting capacity (**Figure 2G**). Correlation analysis within SCCE further revealed that CNV scores were positively associated with EMT but negatively correlated with antigen presentation capacity (**Figure 2H**).

To further investigate functional differences among malignant epithelial cells across pathological subtypes, we conducted GSVA enrichment analysis. SCCE cells exhibited prominent enrichment in pathways associated with mitotic spindle, angiogenesis, and EMT. In contrast, ESCC cells were enriched in immune-linked and metabolic pathways, including the interferon response, the reactive oxygen species (ROS) pathway, DNA repair, the MTORC1 signaling pathway, and oxidative phosphorylation. EAC cells partially overlapped with ESCC in metabolic programs but displayed generally weaker pathway enrichment overall (**Figure 2I**).

To further validate the neuroendocrine (NE) identity of SCCE and investigate relevant regulatory pathways, we calculated an NE signature score using canonical NE markers, including ASCL1, NEUROD1, NKX2-1, INSM1, CHGA, CHGB, NCAM1, and SYP. As shown in **Figure 2J**, SCCE exhibited significantly elevated NE scores compared to EAC and ESCC, reinforcing its distinct small cell–like phenotype. We next assessed the activity of signaling pathways closely associated with NE differentiation and tumor progression, namely the Wnt, Notch, and Hippo pathways. SCCE cells demonstrated markedly increased Wnt signaling activity, but reduced Notch and Hippo pathway activity, relative to the other subtypes. These findings highlight subtype-specific regulatory programs that may contribute to the aggressive biological behavior of SCCE.

### In-depth characterization of molecular heterogeneity in malignant epithelial cells of SCCE

**Figure 3:**
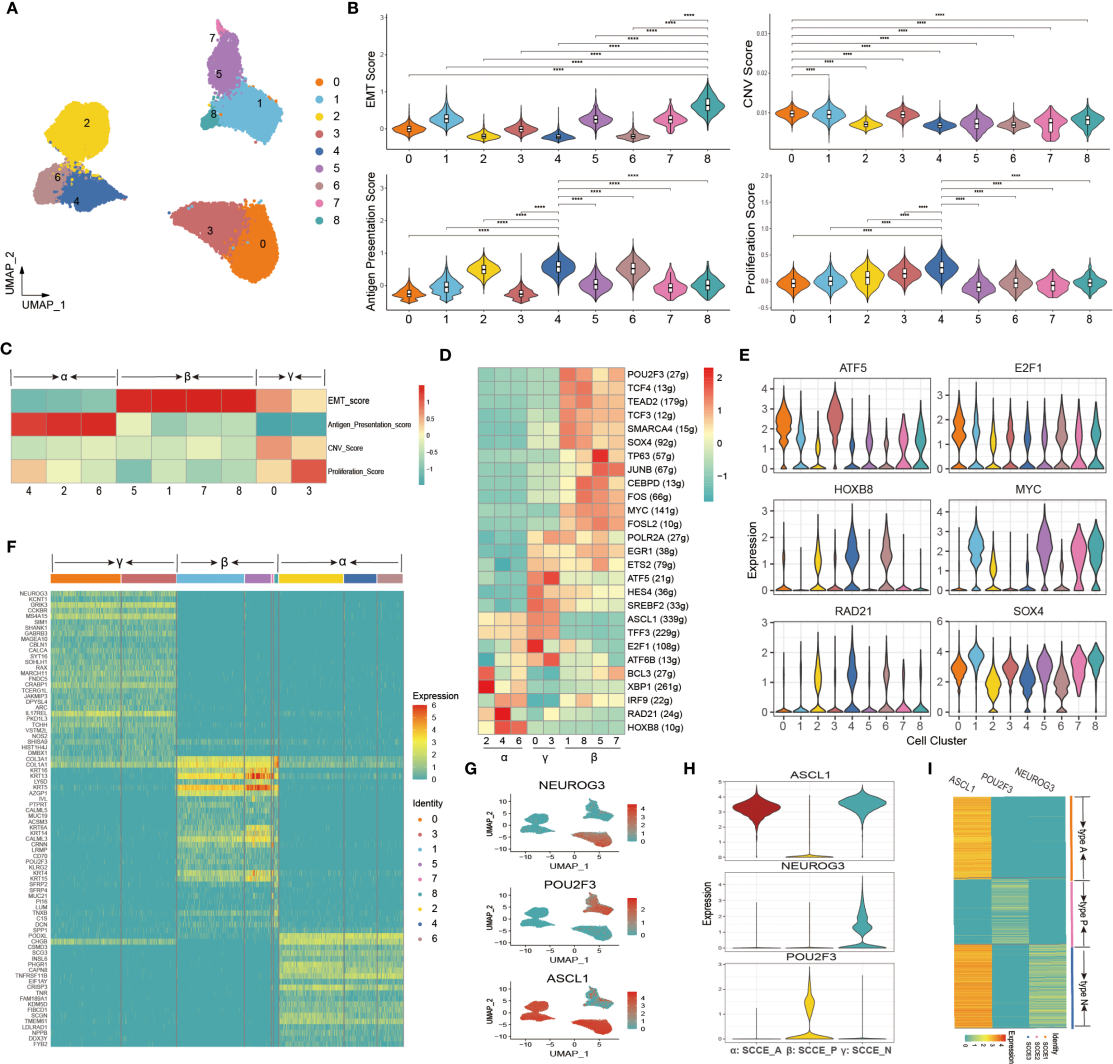
Molecular heterogeneity and transcriptional subtypes of malignant epithelial cells in SCCE. **(A)** UMAP plot of malignant epithelial cells in SCCE, revealing nine transcriptionally distinct subclusters. **(B)** Violin plots showing the distribution of functional scores across the nine subclusters. Four key biological programs were evaluated: EMT, CNV, antigen presentation, and proliferation. Statistical comparisons were performed using the Wilcoxon rank-sum test. ****p < 0.0001. **(C)** Heatmap of functional scores, defining three molecular states: α (clusters 2, 4, 6), β (clusters 1, 5, 7, 8), and γ (clusters 0, 3). **(D)** Heatmap showing the top-ranking TFs distinguishing the three molecular states. **(E)** Violin plots displaying the expression of representative TFs across the nine malignant epithelial subclusters. **(F)** Heatmap of top 10 DEGs in each cluster, supporting the three-state classification. **(G)** UMAP feature plots showing the expression of representative marker genes across malignant epithelial cells. **(H)** Violin plots of NEUROG3, POU2F3, and SCGN expression across subclusters. **(I)** Heatmap displaying NEUROG3, POU2F3, and SCGN expression across individual SCCE samples.

We further dissected the intratumoral heterogeneity of SCCE by focusing on malignant epithelial cells and performing subclustering analysis. UMAP visualization revealed nine distinct transcriptional subpopulations (**Figure 3A**). Functional assessment of each cluster demonstrated significant variation in key biological programs, including EMT, proliferation, CNV, and antigen presentation (**Figure 3B**). A heatmap of four key biological scores revealed distinct functional patterns across clusters, allowing classification into three molecular states: α (clusters 2, 4, 6), β (clusters 1, 5, 7, 8), and γ (clusters 0, 3) (**Figure 3C**). High EMT and moderate proliferation scores characterized the α state; the β state exhibited uniformly high EMT scores but low antigen activity, while the γ state showed elevated proliferation and CNV scores along with the lowest antigen presentation capacity. To further support the functional classification of epithelial subclusters, we examined the expression of canonical NE markers, including ASCL1, NEUROD1, CHGA, NCAM1, and SYP, across all nine epithelial clusters. As shown in **Supplementary Figure 3**, clusters 2, 4, and 6 exhibited markedly elevated NE marker expression, supporting their annotation as NE-positive tumor cells. In contrast, clusters 1, 5, 7, and 8 showed minimal expression of NE markers, consistent with NE-negative phenotypes. Notably, clusters 0 and 3 also demonstrated moderate expression of selected NE markers, suggesting the presence of partial or heterogeneous NE differentiation within the γ state.

We next explored the transcriptional regulatory landscape underlying these subpopulations by analyzing transcription factor (TF) activity. A heatmap of the top-ranking TFs demonstrated state-specific regulatory profiles, clearly distinguishing the α, β, and γ states (**Figure 3D**). Notably, Several Wnt-related TFs such as TP63, JUNB, FOS, and FOSL2 showed preferential activity in the β state. Consistent with this pattern, β state cells exhibited significantly higher Wnt signaling scores than the α and γ states (P < 0.0001; **Supplementary Figure 4**). Violin plots further highlighted representative TFs preferentially active in each state (**Figure 3E**). Consistently, heatmap analysis of the top 10 differentially expressed genes per cluster reaffirmed the presence of three transcriptionally distinct subtypes, each defined by unique signature gene expression patterns (**Figure 3F**).

Based on the transcriptional and regulatory profiles, ASCL1, POU2F3, and NEUROG3 were identified as representative markers of the α, β, and γ states, respectively. These markers exhibited distinct spatial distributions in UMAP space (**Figure 3G**), and violin plots confirmed their subtype-specific expression (**Figure 3H**). Accordingly, we defined three molecular phenotypes of SCCE: SCCE_N (NEUROG3^+^), SCCE_P (POU2F3^+^), and SCCE_A (ASCL1^+^). Finally, we examined gene expression patterns across individual SCCE samples. We found that each patient could be unambiguously classified into one of the three molecular types, indicating inter-patient heterogeneity aligned with the transcriptional subtypes (**Figure 3I**).

### Trajectory analysis reveals distinct differentiation routes and molecular programs of SCCE epithelial cells

**Figure 4:**
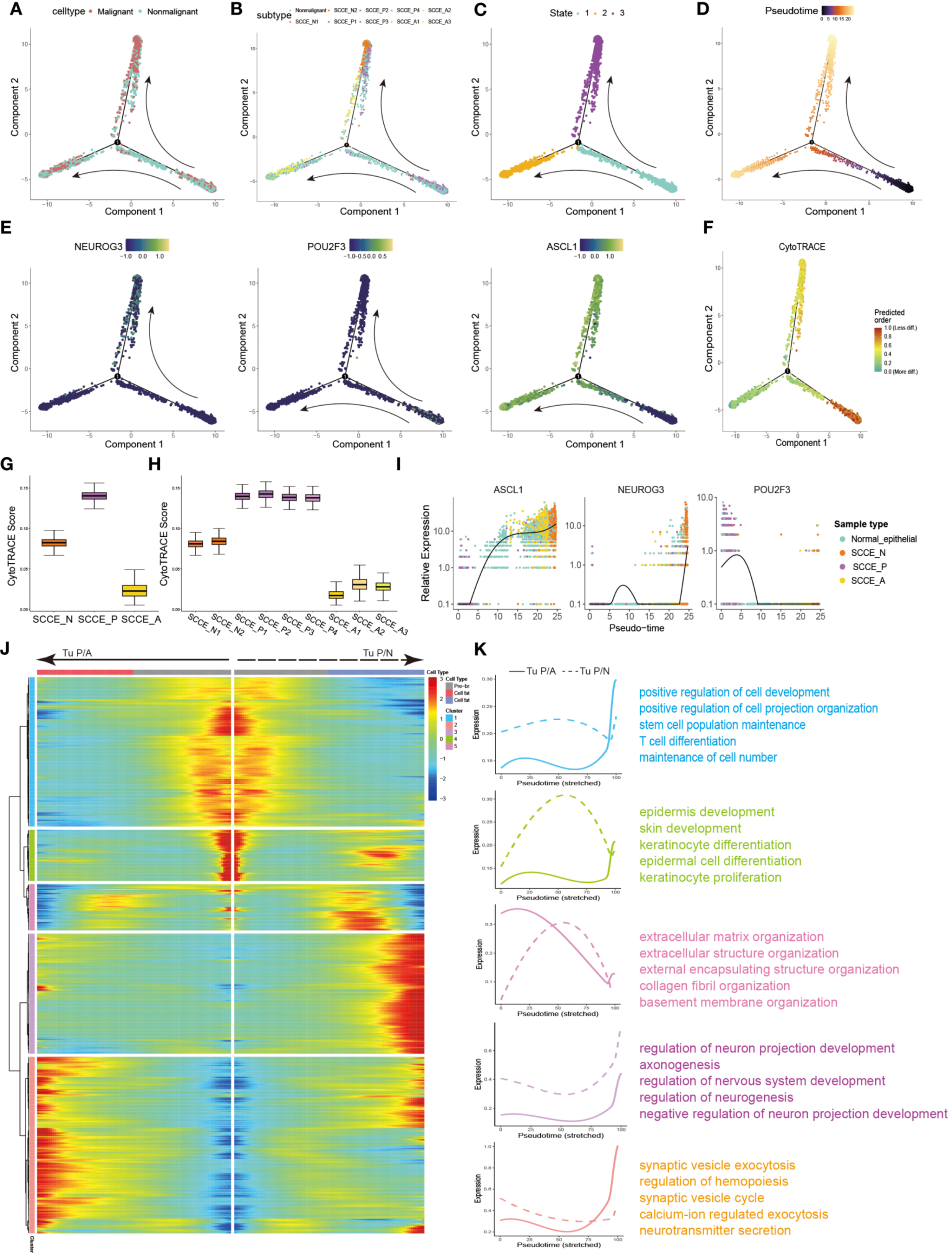
Differentiation trajectories and evolutionary dynamics of SCCE epithelial cells. **(A)** Pseudotime trajectory of SCCE epithelial cells colored by cell type. **(B)** Pseudotime trajectory labeled by 10 epithelial subclusters. **(C)** Cells colored by three pseudotime-defined states. **(D)** Pseudotime trajectory colored by progression along two distinct directions: from SCCE_P toward SCCE_N (Tu P/N) and toward SCCE_S (Tu P/S). **(E)** Pseudotime trajectories of NEUROG3, POU2F3, and SCGN expression. **(F)** Pseudotime trajectory labeled the CytoTRACE scores. **(G)** Box plot of CytoTRACE scores across the three malignant epithelial subtypes. **(H)** Boxplot of CytoTRACE scores across malignant subclusters, grouped by molecular subtype. **(I)** Pseudotime expression trends of NEUROG3, POU2F3, and SCGN across SCCE epithelial cells. **(J)** Heatmap showing gene expression changes along the two tumorigenic trajectories. Color scale (blue to red) indicates increasing expression levels. Genes with similar dynamic patterns were grouped into seven modules. **(K)** Smoothed expression trends along the two evolutionary paths and functional enrichment analysis of the seven gene modules.

To delineate the differentiation hierarchy of SCCE epithelial cells, we performed pseudotime trajectory analysis, including both malignant and non-malignant epithelial subsets. The resulting trajectory exhibited a clear bifurcation, with the SCCE_P cells concentrated at the root, suggesting that this subtype may represent a common progenitor-like population (**Figures 4A, B**). In contrast, SCCE_N and SCCE_A cells occupied distinct terminal branches, corresponding to divergent differentiation trajectories. This pattern was further supported by the distribution of pseudotime states and values (**Figures 4C, D**), where State 1 aligned with SCCE_P, and States 2 and 3 corresponded to SCCE_N and SCCE_A, respectively.

Expression dynamics of key subtype-defining genes along pseudotime further supported this model. POU2F3 was highly expressed at the root, whereas NEUROG3 and ASCL1 exhibited branch-specific upregulation toward the SCCE_N and SCCE_A termini, respectively (**Figure 4E**). Consistently, CytoTRACE analysis revealed the highest differentiation potential in SCCE_P cells, followed by SCCE_N and SCCE_A, indicating progressive maturation along both lineages (**Figures 4F–H**), which suggests a progressive maturation along both trajectories.

We next investigated the dynamics of branch-specific gene expression. NEUROG3 and ASCL1 expression progressively increased along the SCCE_N and SCCE_A branches, respectively, whereas POU2F3 expression declined along both trajectories (**Figure 4I**). Branch-specific gene module analysis revealed distinct transcriptional programs (**Figure 4J**). Genes enriched along the P-to-N trajectory were associated with stemness, cell projection, and T cell differentiation, whereas those along the P-to-S trajectory were linked to epithelial development, extracellular matrix remodeling, and neurodevelopmental processes (**Figure 4K**).

Together, these results suggest that malignant epithelial cells in SCCE originate from a progenitor-like SCCE_P state and diverge along two distinct differentiation trajectories, giving rise to SCCE_N and SCCE_A subtypes with unique transcriptional profiles and functional programs.

### Immune landscape analysis reveals distinctive lymphoid and myeloid remodeling in SCCE

**Figure 5:**
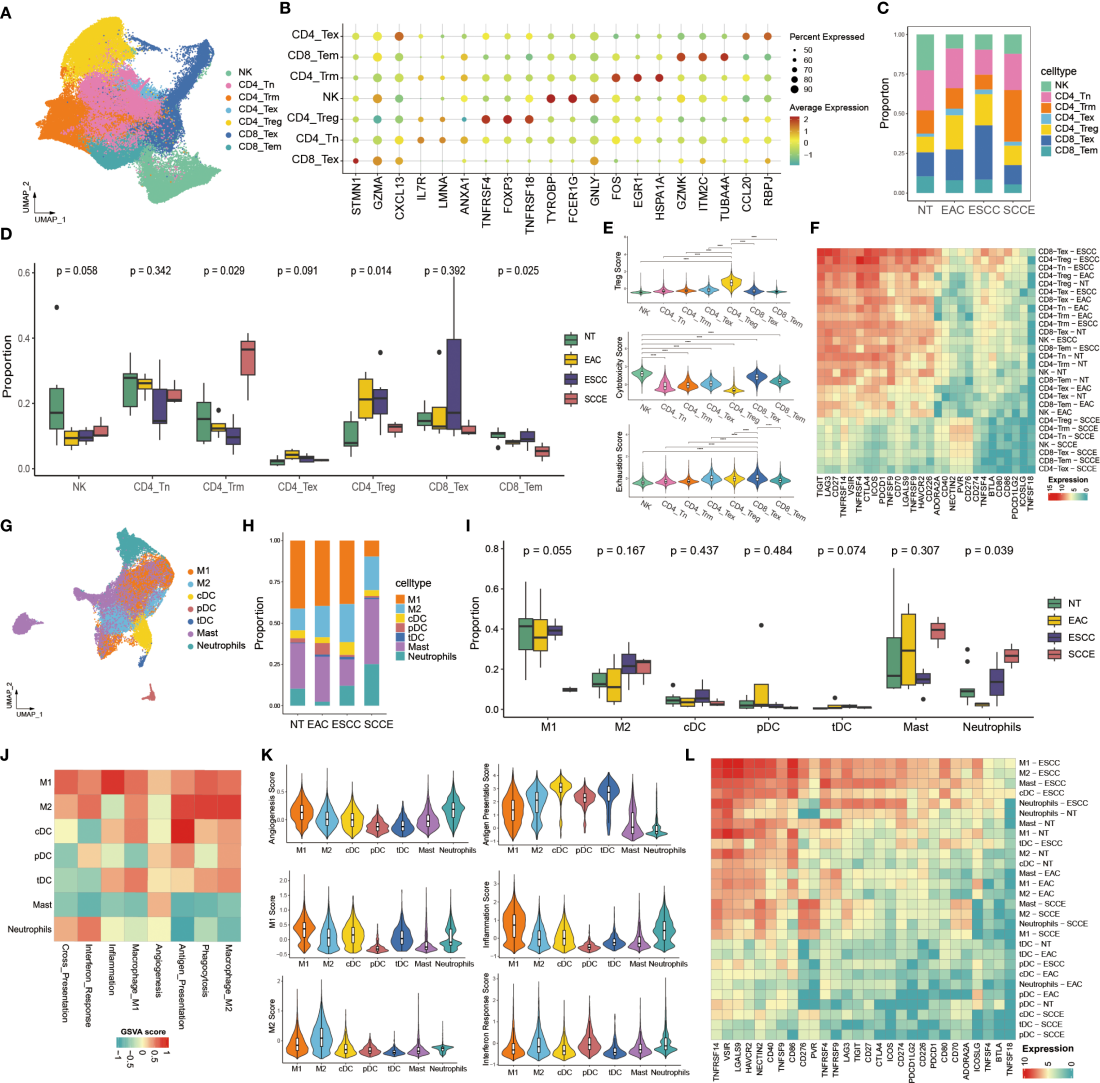
Comprehensive analysis of tumor-infiltrating lymphoid and myeloid cells across esophageal cancer subtypes. **(A)** UMAP visualization of T cells and NK cells reveals eight distinct subpopulations. **(B)** Dot plot showing canonical marker gene expression across lymphoid subsets. **(C)** Stacked bar plot displaying proportional distribution of T/NK subsets across pathological groups. **(D)** Box plots comparing the proportions of NT, EAC, ESCC, and SCCE samples within each lymphoid subset. **(E)** Violin plots showing Treg scores, cytotoxicity scores, and exhaustion scores across subsets. **(F)** Heatmap showing expression of 28 immune checkpoint genes across T/NK subsets by histological group. **(G)** UMAP projection of myeloid cells identifying seven subpopulations, including M1/M2 macrophages, dendritic cells (cDCs, pDCs, tDCs), mast cells, and neutrophils. **(H)** Stacked bar plot showing the distribution of myeloid subsets across pathological subtypes. **(I)** Box plots comparing the proportions of NT, EAC, ESCC, and SCCE samples within each myeloid subset. **(J)** Heatmap showing GSVA scores of representative functional pathways in each myeloid subset. **(K)** Violin plots depicting functional scores across myeloid subsets. **(L)** Heatmap showing expression of 28 immune checkpoint genes across myeloid cell subsets by histological group. *Statistical comparisons in panels **(D, E, I)** were performed using the Wilcoxon rank-sum test. ****p < 0.0001.

To delineate the immune microenvironment across esophageal cancer subtypes, we conducted a detailed analysis of tumor-infiltrating lymphoid and myeloid cells. T/NK cells were clustered into eight transcriptionally distinct subpopulations based on UMAP projection, including CD4_Tn, CD4_Trm, CD4_Tex, CD4_Treg, CD8_Tex, CD8_Tem, and NK cells (**Figure 5A**). Signature markers for each subset, such as FOXP3 (CD4_Treg), GZMK (CD8_Tem), and CXCL13 (CD4_Tex), were confirmed by dot plot analysis (**Figure 5B**). The distribution of these subsets varied across pathological groups. Notably, CD4_Trm cells were markedly enriched in SCCE compared to other histological types and represented the most abundant T/NK subset in this group (**Figure 5C**). Quantitative comparisons further confirmed a significantly higher proportion of CD4_Trm cells in SCCE (p = 0.029), whereas CD4_Treg (p = 0.014) and CD8_Tem cells (p = 0.025) were relatively decreased (**Figure 5D**). In terms of functional states, CD4_Treg cells exhibited elevated regulatory scores, NK cells showed enhanced cytotoxic activity, and CD8_Tex cells demonstrated pronounced exhaustion (**Figure 5E**). Expression profiling of 28 commonly studied immune checkpoint genes revealed a broad downregulation in SCCE, with most checkpoint ligands and receptors expressed at low levels across nearly all T/NK subsets. In contrast, ESCC samples exhibited widespread upregulation of immune checkpoints (**Figure 5F**).

In the analysis of myeloid cells, UMAP projection revealed seven distinct subpopulations: M1 and M2 macrophages, conventional and plasmacytoid dendritic cells (cDCs and pDCs), tolerogenic dendritic cells (tDCs), mast cells, and neutrophils (**Figure 5G**). Compositional analysis showed a marked decrease in M1 macrophages and a substantial increase in mast cells and neutrophils in SCCE relative to other pathological groups, with mast cells constituting the most abundant subset in SCCE (**Figure 5H**). Although intergroup comparisons did not reach statistical significance (**Figure 5I**), the observed compositional trends were consistent with those noted in **Figure 5H**. Functional profiling of myeloid subsets revealed strong enrichment of multiple immunological pathways in macrophages, particularly in M1-like cells, including pathways associated with inflammation, antigen presentation, and interferon responses (**Figure 5J**). Violin plots further illustrated distinct functional patterns among subsets, with elevated M1 and inflammation scores in M1 macrophages and increased antigen presentation capacity in cDCs (**Figure 5K**). Immune checkpoint expression in myeloid cells mirrored the pattern observed in lymphoid cells, featuring overall downregulation in SCCE and relatively higher expression levels in ESCC samples across multiple subtypes (**Figure 5L**).

### Fibroblast heterogeneity and ELF3-associated transcriptional features in the SCCE stroma

**Figure 6:**
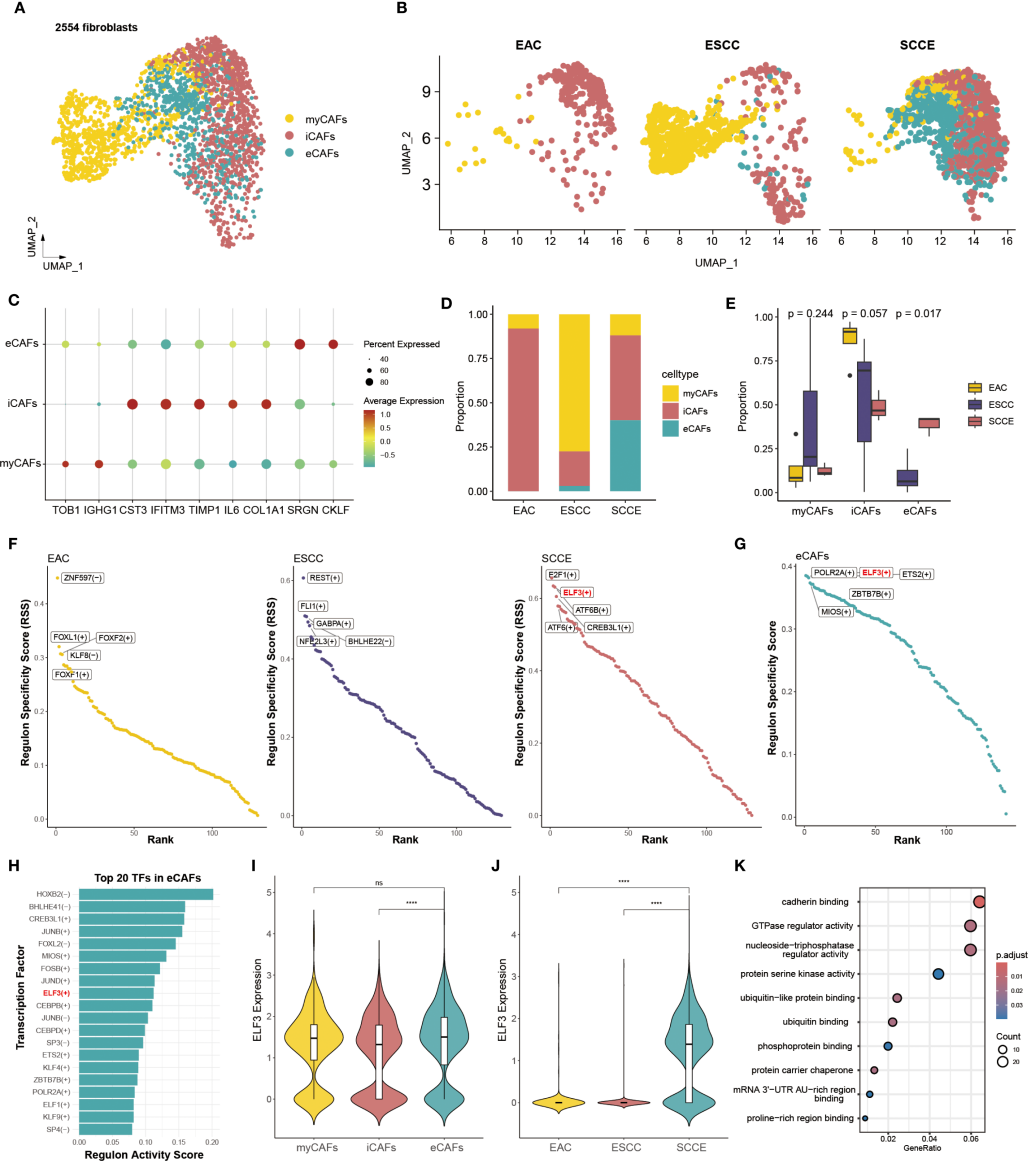
SCCE-specific enrichment of eCAFs and associated regulatory activity of ELF3. **(A)** UMAP plot showing the clustering of all fibroblast cells into three subtypes. **(B)** UMAP plots displaying the distribution of fibroblast subtypes across EAC, ESCC, and SCCE samples. **(C)** Dot plot showing the expression of representative marker genes for each fibroblast subtype. **(D)** Stacked bar plot showing the relative abundance of each fibroblast subtype across cancer types. **(E)** Box plots comparing the proportion of EAC, ESCC, and SCCE samples within each fibroblast subtypes. Statistical comparisons were performed using the Wilcoxon rank-sum test. **(F)** Scatter plots of RSS for transcription factors in EAC, ESCC, and SCCE, with top five subtype-specific regulators labeled. **(G)** TFs in eCAFs ranked by RSS, with the top five regulators labeled. **(H)** Top 20 TFs in eCAFs ranked by RAS. **(I)** Violin plot showing ELF3 expression across the three fibroblast subtypes. **(J)** Violin plot showing ELF3 expression across cancer types. **(K)** KEGG pathway enrichment analysis of ELF3 target genes, with top 10 pathways shown.

Unsupervised clustering identified three distinct fibroblast subtypes, namely myofibroblastic CAFs (myCAFs), inflammatory CAFs (iCAFs), and extracellular matrix CAFs (eCAFs), as visualized by UMAP projection (**Figure 6A**). These subtypes displayed distinct distribution patterns across cancer types (**Figures 6B–D**). eCAFs were absent in EAC, barely detectable in ESCC, but significantly enriched in SCCE. iCAFs were present in all three groups, with the highest proportion in EAC, whereas myCAFs predominated in ESCC. Comparative analysis confirmed that eCAFs comprised a significantly higher fraction of fibroblasts in SCCE compared to ESCC and EAC (p = 0.017), highlighting a disease-specific expansion (**Figure 6E**).

Differentially expressed genes were identified across the three CAF subtypes. The top 20 representative markers demonstrated clear separation among myCAFs, iCAFs, and eCAFs, reflecting distinct molecular signatures (**Supplementary Figure 5A**). GO enrichment analysis indicated functional specialization. myCAFs were enriched for gene sets related to RNA splicing and mRNA processing (**Supplementary Figure 5B**). iCAFs showed upregulation of genes involved in extracellular matrix organization, collagen metabolism, and cell–substrate adhesion (**Supplementary Figure 5C**). eCAFs were characterized by signatures associated with mitotic division, cell cycle checkpoint control, and chromatid segregation, suggesting a proliferative phenotype (**Supplementary Figure 5D**). Expression levels of six classical CAF markers were also examined. CD248, a mechanoresponsive fibroblast marker associated with immune exclusion and therapy resistance, showed predominant expression in iCAFs, along with COL1A1, POSTN, and DCN. ACTA2 was mainly expressed in eCAFs and iCAFs, while IL6 remained low across all subtypes (**Supplementary Figure 6**).

Transcription factor analysis based on regulatory specificity scores (RSS) revealed distinct transcriptional programs in CAFs across cancer subtypes. The top five transcription factors ranked by RSS were entirely non-overlapping between EAC, ESCC, and SCCE, indicating highly divergent regulatory landscapes (**Figure 6F**). In SCCE, the highest-ranking transcription factors included E2F1, ELF3, ATF6B, CREB3L1, and ATF6. Within the eCAF subset, ELF3 also emerged among the top five transcription factors based on RSS (**Figure 6G**). Moreover, ELF3 ranked within the top 20 regulators by transcriptional activity scores (RAS) in eCAFs (**Figure 6H**), further highlighting its prominence. These findings prompted closer examination of ELF3 expression, which was significantly elevated in eCAFs compared to other CAF subtypes (**Figure 6I**), and most pronounced in SCCE samples relative to other histological types (**Figure 6J**). Functional enrichment analysis of ELF3 target genes revealed associations with cancer-related pathways, including cadherin binding, regulation of GTPase activity, and kinase signaling, underscoring its potential role in fibroblast-mediated tumor behavior (**Figure 6K**).

### SCCE exhibits distinct intercellular communication patterns dominated by collagen signaling and iCAF-mediated interactions

**Figure 7:**
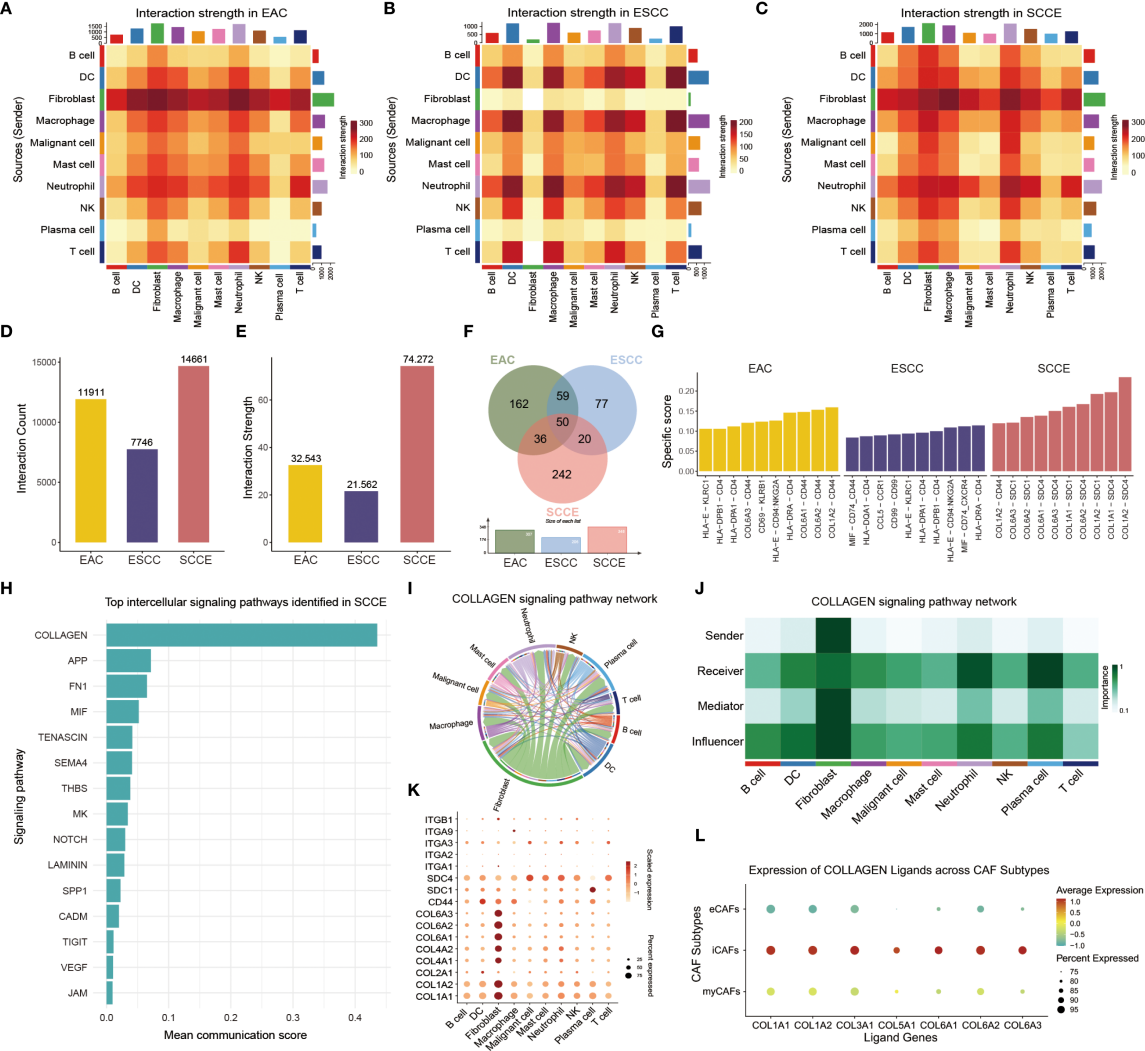
Intercellular communication landscape and collagen signaling features in SCCE. **(A–C)** Heatmaps showing intercellular interaction strength among ten major cell types in EAC **(A)**, ESCC **(B)**, and SCCE **(C)**. **(D, E)** Bar plots comparing the total number of ligand-receptor pairs **(D)** and cumulative interaction strength **(E)** in each subtype. **(F)** Venn diagram illustrating unique and shared ligand–receptor interactions among the three subtypes. **(G)** Top ten ligand-receptor pairs in each cancer subtype ranked by subtype specificity score. **(H)** Bar plot displaying the top 15 signaling pathways ranked by communication strength in SCCE. **(I)** Circle plot showing the direction and magnitude of interactions between cell populations within the collagen signaling pathway in SCCE. **(J)** Heatmap of network centrality scores for each cell type in SCCE collagen signaling. **(K)** Dot plot showing expression of collagen pathway–associated ligands and receptors across major cell types in SCCE. **(L)** Dot plot of collagen ligand expression in three CAF subtypes.

To explore intercellular communication patterns across esophageal cancer subtypes, we performed a comprehensive analysis of cell-cell interactions encompassing ten major cell lineages: malignant epithelial cells, fibroblasts, T cells, B cells, NK cells, plasma cells, macrophages, dendritic cells, mast cells, and neutrophils. Heatmaps depicting interaction strength revealed distinct subtype-specific signaling landscapes: EAC and ESCC exhibited relatively focused interaction patterns, whereas SCCE showed more widespread and uniformly distributed intercellular signaling (**Figures 7A–C**). Quantitatively, SCCE demonstrated the highest number of interactions and the greatest cumulative interaction strength among the three groups (**Figures 7D, E**). A comparative analysis of ligand-receptor pairs revealed that SCCE exhibited the largest number of total interactions, while only 50 pairs were shared across all histological types, underscoring the divergent communication networks among the subtypes (**Figure 7F**). The top ten ligand–receptor interactions ranked by subtype-specific scores showed minimal overlap, with SCCE-enriched interactions predominantly involving multiple combinations of COL6A/COL1A ligands and SDC1/SDC4 receptors, indicating a distinct ligand–receptor signature in this subtype (**Figure 7G**).

Pathway-level analysis further identified the collagen signaling cascade as the most prominent intercellular communication axis in SCCE, followed by APP, FN1, and MIF signaling (**Figure 7H**). Within the collagen pathway, fibroblasts emerged as the dominant mediators, with extensive outgoing and incoming interactions involving multiple immune and stromal cell types, as visualized by a circle plot and a network centrality heatmap (**Figures 7I, J**). Further inspection of collagen-related ligand–receptor pairs across cell types revealed distinct expression patterns, particularly within fibroblasts. (**Figure 7K**). Stratified analysis of CAF subtypes revealed that iCAFs displayed the highest average expression and the broadest coverage of collagen ligands, whereas eCAFs exhibited markedly lower expression levels. These findings suggest that iCAFs may represent the predominant source of extracellular matrix components involved in collagen-mediated intercellular signaling (**Figure 7L**).

## Discussion

Given the rarity of SCCE, single-cell transcriptomic studies remain extremely limited. To date, only two such studies have been reported. One analyzed treatment-naive and post-chemotherapy tumor samples from only a single SCCE patient, focusing on therapy-induced changes (26). The other, more recent study profiled SCCE tumors and reported key differences in immune infiltration and epithelial lineage (9). While these findings represent important early efforts, our understanding of the tumor-intrinsic heterogeneity and microenvironmental interactions in SCCE remains incomplete.

In this study, we performed an integrative single-cell transcriptomic analysis of SCCE in comparison with EAC, ESCC, and NT, aiming to construct a comprehensive cellular atlas of tumor and microenvironmental heterogeneity. SCCE tumors exhibited a distinct cellular architecture characterized by increased epithelial cell fractions, reduced immune infiltration, and widespread genomic instability. Malignant epithelial cells displayed substantial transcriptional heterogeneity, forming three subtypes with divergent differentiation trajectories. At the microenvironmental level, SCCE was marked by global downregulation of immune checkpoint genes, eCAF expansion associated with upregulation of the transcription factor ELF3, and intensified collagen signaling predominantly driven by iCAFs. Collectively, these findings delineate the unique tumor ecosystem of SCCE and highlight its molecular divergence from other esophageal cancer subtypes.

A defining feature of malignant epithelial cells in SCCE was pronounced genomic instability, characterized by widespread chromosomal amplifications and deletions, a pattern also observed in other neuroendocrine carcinomas (27). Interestingly, SCCE samples exhibited a bimodal distribution of CNV scores, in contrast to the unimodal patterns observed in other subtypes, suggesting the presence of subclonal structures with varying degrees of genomic alteration. These findings suggest that CNV heterogeneity may contribute to the molecular and phenotypic intratumoral diversity observed in SCCE. Functionally, higher CNV scores were positively associated with EMT and proliferation signatures, linking genomic instability to more aggressive tumor behavior, as reported in various malignancies where elevated CNV burden correlates with invasiveness and poor prognosis (28, 29). These genomic alterations not only reflect extensive intratumoral heterogeneity but may also help explain the aggressive clinical behavior of SCCE, including its rapid proliferation and early metastatic potential. Moreover, this genomic instability may underlie the transcriptional divergence observed among malignant subpopulations, consistent with the notion that genomic alterations can drive phenotypic diversification in cancer (30).

Building on the pronounced transcriptional heterogeneity observed in SCCE epithelial cells, we identified three malignant subtypes: SCCE_N (NEUROG3^+^), SCCE_P (POU2F3^+^), and SCCE_A (ASCL1^+^), each defined by distinct transcription factor activity and functional phenotypes. These subtypes exhibited distinct activity across key biological programs, including EMT, proliferation, and antigen presentation, indicating functional diversification beyond conventional histological classification. Our molecular stratification parallels and expands the subtype frameworks previously established in SCLC, where malignant epithelial cells were classified into A-type (ASCL1^+^), N-type (NEUROD1^+^), and P-type (POU2F3^+^) groups (31, 32). Molecular subtypes defined by lineage-specific transcription factors have also been identified in small cell carcinomas of the bladder (33), prostate (34), and cervix (35). In our study, although the SCCE_P and SCCE_A subtypes resembled the P-type and A-type defined in SCLC, the SCCE_N subtype displayed a unique transcriptional profile not previously reported in small cell carcinomas. These observations point to both shared and lineage-specific regulatory programs among small cell malignancies arising from different tissue origins. Although the clinical implications of SCCE molecular subtypes remain to be established, their distinct transcriptional identities provide a foundation for future mechanistic investigation and biological characterization.

To further dissect the developmental hierarchy of SCCE epithelial cells, we performed pseudotime trajectory analysis incorporating both malignant and non-malignant populations. The analysis revealed a bifurcated lineage structure, with SCCE_P cells localized at the trajectory root and exhibiting the highest CytoTRACE scores, consistent with a progenitor-like state. This observation aligns with prior reports describing P-type cells as early progenitors in SCLC (36). In contrast, SCCE_N and SCCE_A cells were distributed along two terminal branches, each associated with distinct gene expression programs. NEUROG3, a bHLH transcription factor essential for pancreatic endocrine cell fate and known to cooperate with NEUROD1 in α-cell differentiation, emerged as a defining marker of SCCE_N. This suggests a neuroendocrine differentiation route distinct from the N-type subtype commonly described in SCLC (37). Meanwhile, the SCCE_A subtype was enriched for pathways related to extracellular matrix organization and neurodevelopment, potentially representing a more differentiated, stroma-interacting phenotype. ASCL1 has served as a defining marker for molecular subtyping in multiple small cell carcinomas, reflecting its central role in neuroendocrine differentiation (31, 35). Together, these findings suggest that SCCE molecular subtypes not only capture static transcriptional identities but also reflect distinct differentiation trajectories, with implications for tumor plasticity, therapeutic resistance, and lineage-specific vulnerabilities.

To address the limited number of SCCE samples analyzed, we explicitly considered the magnitude and consistency of transcriptional differences across the identified malignant subtypes. Despite the small cohort, the subtypes SCCE_P, SCCE_N, and SCCE_A exhibited substantial divergence across multiple independent analyses. These included distinct functional signatures such as EMT, proliferation, and antigen presentation; differences in TF activity measured by RSS and RAS; and separation along pseudotime-defined differentiation trajectories. The reproducibility of these findings across orthogonal modalities suggests that the observed subtype distinctions are unlikely to be random artifacts, but rather reflect true biological heterogeneity. While we acknowledge that the small sample size limits the statistical certainty of our conclusions, the observed effect sizes provide preliminary support for the biological relevance of this classification.

Nevertheless, we acknowledge that the small sample size and the absence of experimental validation models inherently limit the generalizability of our findings. While the identified subtypes demonstrate strong internal consistency, the conclusions drawn may still be influenced by sampling bias or inter-patient variability. In addition, without supporting evidence from *in vitro* or *in vivo* models, it remains uncertain whether the observed transcriptional states represent stable phenotypic identities or transient transcriptional programs. Future studies involving larger patient cohorts and functional assays will be crucial to confirm the biological relevance and clinical significance of the SCCE molecular subtypes proposed in this study.

Beyond tumor-intrinsic alterations, our study revealed a profoundly immunosuppressive microenvironment in SCCE. Compared to ESCC and EAC, SCCE tumors exhibited markedly reduced infiltration of both lymphoid and myeloid compartments, consistent with an immune-excluded phenotype. Expression of immune checkpoint genes such as PDCD1, CTLA4, LAG3, and TIGIT was broadly suppressed across SCCE-infiltrating T cells. While the presence of these checkpoints is often associated with T cell exhaustion and may predict responsiveness to ICIs, their marked downregulation in SCCE suggests a lack of pre-existing immune activation. This transcriptomic pattern may reflect an immunologically ‘cold’ tumor phenotype, which has been associated with limited response to checkpoint blockade in other malignancies (38). The above observations were further supported by functional scoring of immune cell subsets, as well as GSVA analysis demonstrating global suppression of antigen presentation, T cell activation, and interferon-related pathways in SCCE. Our findings are consistent with a recent single-cell study of SCCE, which similarly reported an immunosuppressive landscape enriched for regulatory T cells and angiogenesis-associated stromal niches, particularly within a unique tumor microenvironment (TME) ecotype highly enriched in SCCE patients (9). Taken together, these results support the notion that SCCE harbors a fundamentally distinct tumor-immune ecosystem compared to other esophageal cancer subtypes.

Previous transcriptomic studies based on bulk RNA sequencing have suggested a suppressive immune microenvironment in SCCE, typically characterized by M2 macrophage enrichment and limited T cell activation (39). However, due to the lack of cellular resolution, these approaches were unable to resolve cell type specific alterations. In contrast, our single-cell analysis provided a high-resolution view of the SCCE tumor microenvironment. Compared to other esophageal cancer subtypes, SCCE exhibited widespread downregulation of immune checkpoint genes across both lymphoid and myeloid compartments, indicative of pervasive immune dysfunction. Among lymphoid populations, SCCE showed a marked reduction in cytotoxic CD8^+^ T cells, along with skewed CD4^+^ subset distributions, suggesting a compromised adaptive immune response. Myeloid populations were similarly affected, with reduced representation of M1-like macrophages and dendritic cells, and a relative increase in mast cells and neutrophils, indicative of a pro-tumor inflammatory state. Altogether, these features collectively point to a suppressed and functionally imbalanced immune microenvironment in SCCE, in contrast to the more immune-active landscapes observed in ESCC. While direct clinical data on SCCE response to ICIs remain scarce, the transcriptomic profile is characterized by checkpoint downregulation, impaired antigen presentation, and diminished cytotoxicity. These features collectively indicate a low-immunogenic tumor microenvironment. Similar immune phenotypes have been associated with resistance to immune checkpoint inhibitors in multiple other cancers (40), and may partially explain the limited efficacy of ICIs in SCCE. However, further experimental and clinical validation is needed to confirm this association.

In addition to immune alterations, our study revealed notable stromal heterogeneity in SCCE, particularly among CAFs. Among the identified CAF subtypes, eCAFs were significantly enriched in SCCE relative to other esophageal cancer subtypes. Transcription factor analysis identified ELF3 as one of the top-ranked regulons in eCAFs, implicating it in the regulatory programs of this subtype. ELF3 is a multifunctional transcription factor known to regulate epithelial differentiation, EMT, and immune responses in various malignancies. In colorectal and cholangiocarcinoma models, ELF3 has been shown to modulate cancer cell plasticity and maintain epithelial barrier integrity (41, 42). However, its role in stromal compartments remains largely unexplored. Our findings suggest that ELF3 ELF3 may contribute to extracellular matrix remodeling and fibroblast-mediated functions in SCCE, representing a potential regulatory node in the tumor stroma. Additionally, CD248 showed predominant expression in iCAFs. This marker was recently characterized as a feature of mechanoresponsive CAFs that promote immune exclusion and therapeutic resistance in ESCC, based on single-cell transcriptomic analysis in the context of neoadjuvant immunotherapy (43).

Building on these findings, we next examined cell–cell communication and found that SCCE exhibited the most complex interaction network among the esophageal cancer subtypes, characterized by a greater number of interactions, higher interaction strength, and a largely unique ligand–receptor repertoire. This signaling complexity is reminiscent of the interaction-rich microenvironments observed in other CAF-dense tumors (44, 45). Within SCCE, CAFs emerged as the predominant signal-sending population, aligning with their abundance and potential stromal regulatory role. Interestingly, although eCAFs were specifically enriched in SCCE, it was the iCAFs subtype that contributed most significantly to outgoing signaling activity, particularly through the collagen signaling pathway. This finding may reflect the broader paracrine function of inflammatory CAFs, which have been implicated in promoting tumor progression in other cancers via cytokine-mediated activation of NF-κB and STAT3 signaling (46, 47). These observations suggest a division of labor among CAF subtypes in SCCE, with eCAFs potentially driving extracellular matrix remodeling and iCAFs actively contributing to cell–cell communication through paracrine signaling pathways such as collagen.

By delineating the cellular architecture of SCCE at single-cell resolution, our study reveals multiple layers of tumor heterogeneity with potential clinical relevance. The identification of distinct malignant subtypes with divergent transcriptional programs and differentiation trajectories suggests that SCCE comprises biologically diverse tumor cell populations, which may respond differently to treatment. In the immune compartment, the immune-excluded phenotype, characterized by low cytotoxic T cell infiltration and suppressed checkpoint expression, may contribute to poor immunotherapy responsiveness. Furthermore, the expansion of eCAFs and the active paracrine signaling role of iCAFs via collagen pathways highlight stromal components as potential modulators of tumor progression and immune evasion. Collectively, our findings provide a single-cell framework that defines the cellular diversity and immune suppression in SCCE, offering a foundation for further mechanistic investigation and hypothesis-driven research.

Acknowledging the limitations of this study is necessary. First, the rarity of SCCE poses significant challenges in acquiring sufficient specimens, which in turn limits opportunities for experimental validation of key findings, such as chromosome 19 amplifications and molecular subtype classification. Second, the absence of established *in vitro* or *in vivo* models for this rare tumor type precluded experimental validation of the regulatory mechanisms identified, such as ELF3-mediated activation of cancer-associated fibroblasts. Developing experimental model representative of SCCE will be critical for future mechanistic studies. Third, although the proposed molecular subtypes exhibited distinct biological features, their prognostic relevance could not be evaluated due to the lack of sufficiently large patient cohorts for robust survival analyses.

## Conclusion

This study presents a comprehensive single-cell atlas of SCCE, uncovering its distinct tumor architecture, molecular subtypes, and reprogrammed tumor microenvironment. These findings advance our understanding of this rare malignancy and provide a framework for future mechanistic studies and exploratory biological research.

## Data Availability

Publicly available datasets analyzed in this study can be accessed from the GEO database under accession numbers GSE145370 and GSE222078. Raw and processed sequencing data generated in this study have been deposited in the Gene Expression Omnibus (GEO) under accession number GSE308736.

## Supplementary material

Supplementary Figure 1 UMAP visualization before and after integration to correct batch effects across datasets. **(A)** Pre-integration UMAP shows clear separation of cells from SCCE, ESCC (GSE145370), and EAC (GSE222078), reflecting potential batch effects. **(B)** Post-integration UMAP demonstrates effective alignment of cells across the three datasets, indicating successful mitigation of technical noise.

Supplementary Figure 2 Histological comparison of three esophageal cancer subtypes. Representative hematoxylin and eosin (H&E) staining images of EAC, ESCC, and SCCE, highlighting the distinct histomorphological features of each subtype.

Supplementary Figure 3 Expression of NE markers across malignant epithelial cells of SCCE. Violin plots showing the expression of five canonical neuroendocrine markers (ASCL1, NEUROD1, CHGA, NCAM1, and SYP) across nine malignant epithelial clusters.

Supplementary Figure 4 Wnt signaling activity across SCCE molecular subtypes. Violin plots showing Wnt signaling scores across the α, β, and γ subtypes of malignant epithelial cells. Wnt scores were calculated using the AddModuleScore method. *Statistical comparison was performed using the Wilcoxon rank-sum test. ****p < 0.0001.

Supplementary Figure 5 Transcriptomic and functional differences among CAF subtypes. **(A)** Bubble plot showing the top 20 marker genes distinguishing myCAFs, iCAFs, and eCAFs. Dot size indicates the percentage of cells expressing the gene, and color represents average expression level. **(B–D)** GO enrichment analysis of differentially expressed genes in myCAFs **(B)**, iCAFs **(C)**, and eCAFs **(D)**. Top enriched biological processes (BP) are displayed. Dot size represents the number of genes involved, and color denotes adjusted p-values.

Supplementary Figure 6 Expression patterns of canonical CAF markers across fibroblast subtypes. Violin plots showing the expression patterns of six canonical CAF-associated markers (ACTA2, CD248, COL1A1, DCN, IL6, and POSTN) across the three CAF subtypes identified in SCCE.
