# Supplementary figures and images for: Single-cell transcriptomic analysis reveals epithelial and microenvironmental heterogeneity in small cell carcinoma of the esophagus
Source: Front Immunol. 2025 Oct 8;16:1672587. doi: 10.3389/fimmu.2025.1672587 (PMC12540368; doi:10.3389/fimmu.2025.1672587)

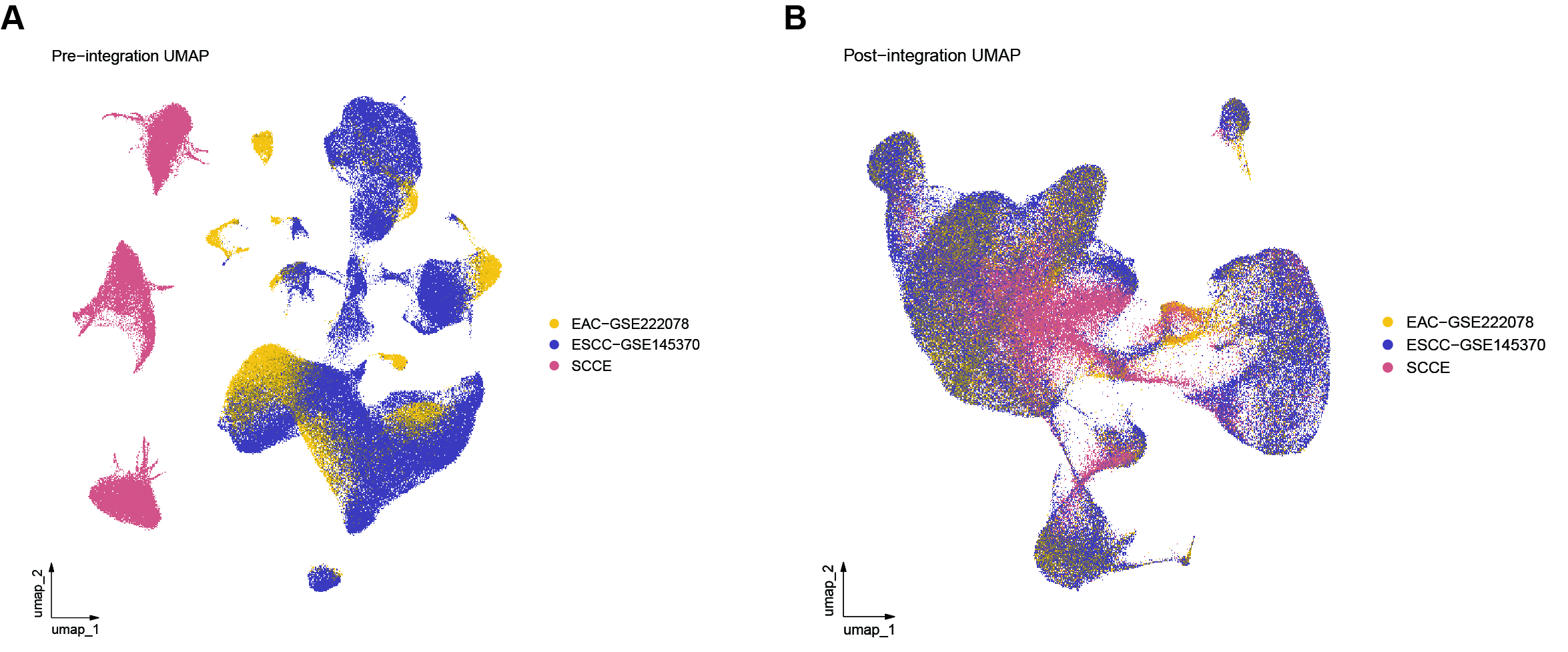

Supplement: Supplementary Figure 1 — UMAP visualization before and after integration to correct batch effects across datasets. (A) Pre-integration UMAP shows clear separation of cells from SCCE, ESCC (GSE145370), and EAC (GSE222078), reflecting potential batch effects. (B) Post-integration UMAP demonstrates effective alignment of cells across the three datasets, indicating successful mitigation of technical noise. [file Image1.tif]

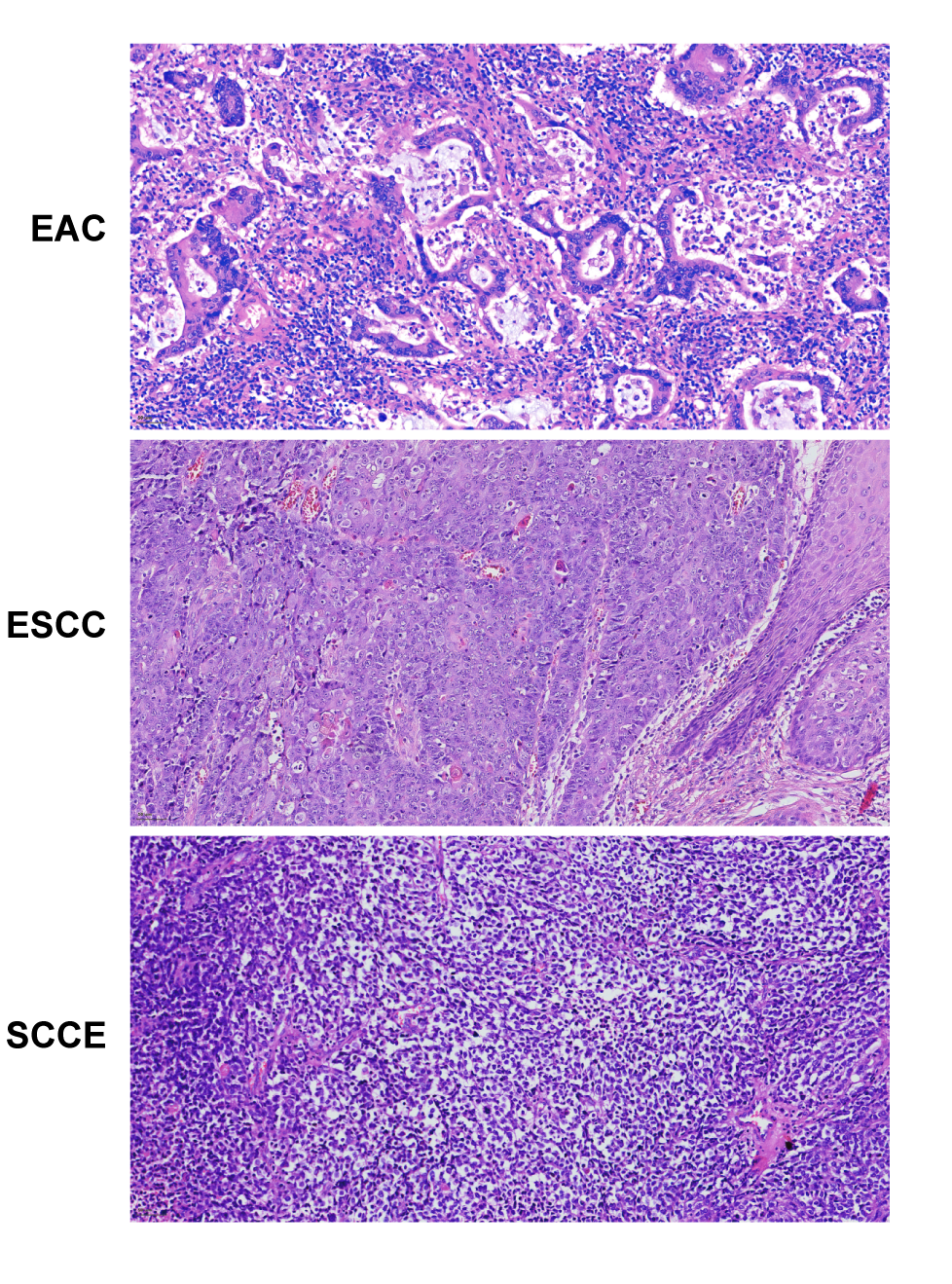

Supplement: Supplementary Figure 2 — Histological comparison of three esophageal cancer subtypes. Representative hematoxylin and eosin (H&E) staining images of EAC, ESCC, and SCCE, highlighting the distinct histomorphological features of each subtype. [file Image2.tif]

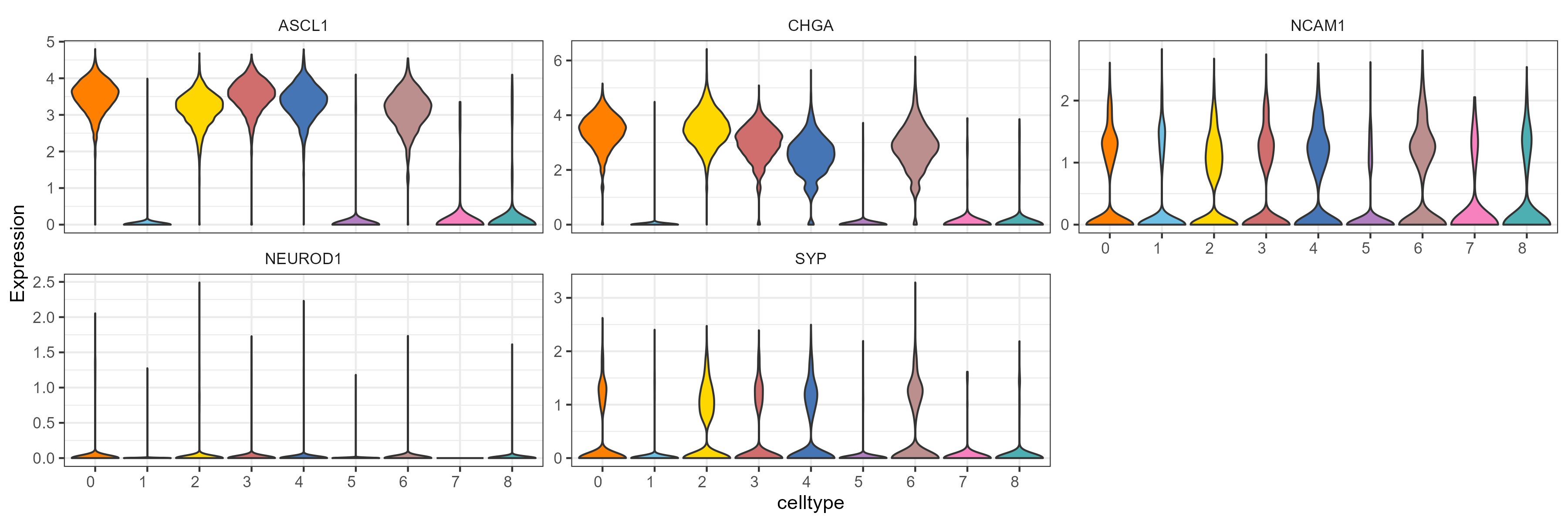

Supplement: Supplementary Figure 3 — Expression of NE markers across malignant epithelial cells of SCCE. Violin plots showing the expression of five canonical neuroendocrine markers (ASCL1, NEUROD1, CHGA, NCAM1, and SYP) across nine malignant epithelial clusters. [file Image3.tif]

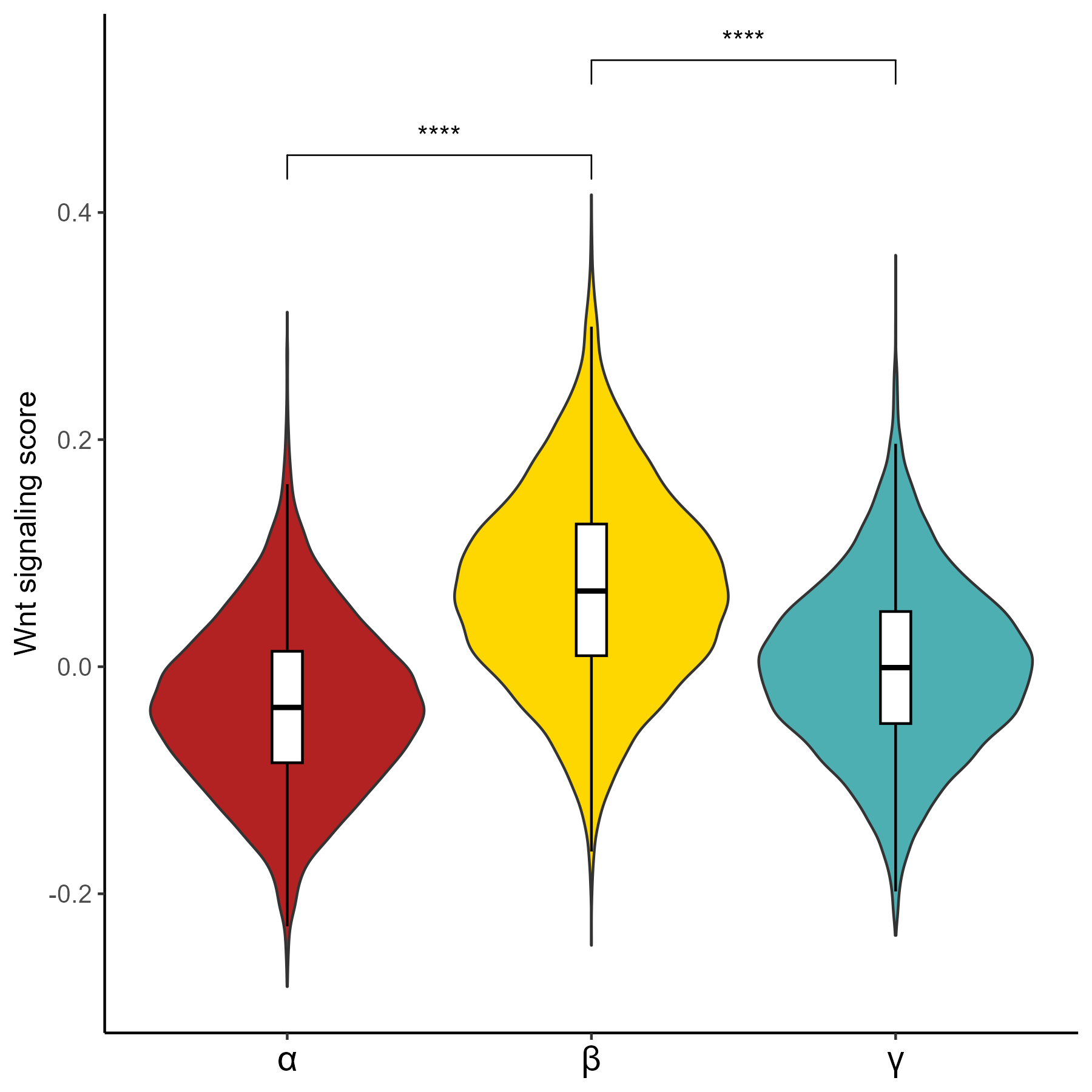

Supplement: Supplementary Figure 4 — Wnt signaling activity across SCCE molecular subtypes. Violin plots showing Wnt signaling scores across the α, β, and γ subtypes of malignant epithelial cells. Wnt scores were calculated using the AddModuleScore method. *Statistical comparison was performed using the Wilcoxon rank-sum test. ****p < 0.0001. [file Image4.tif]

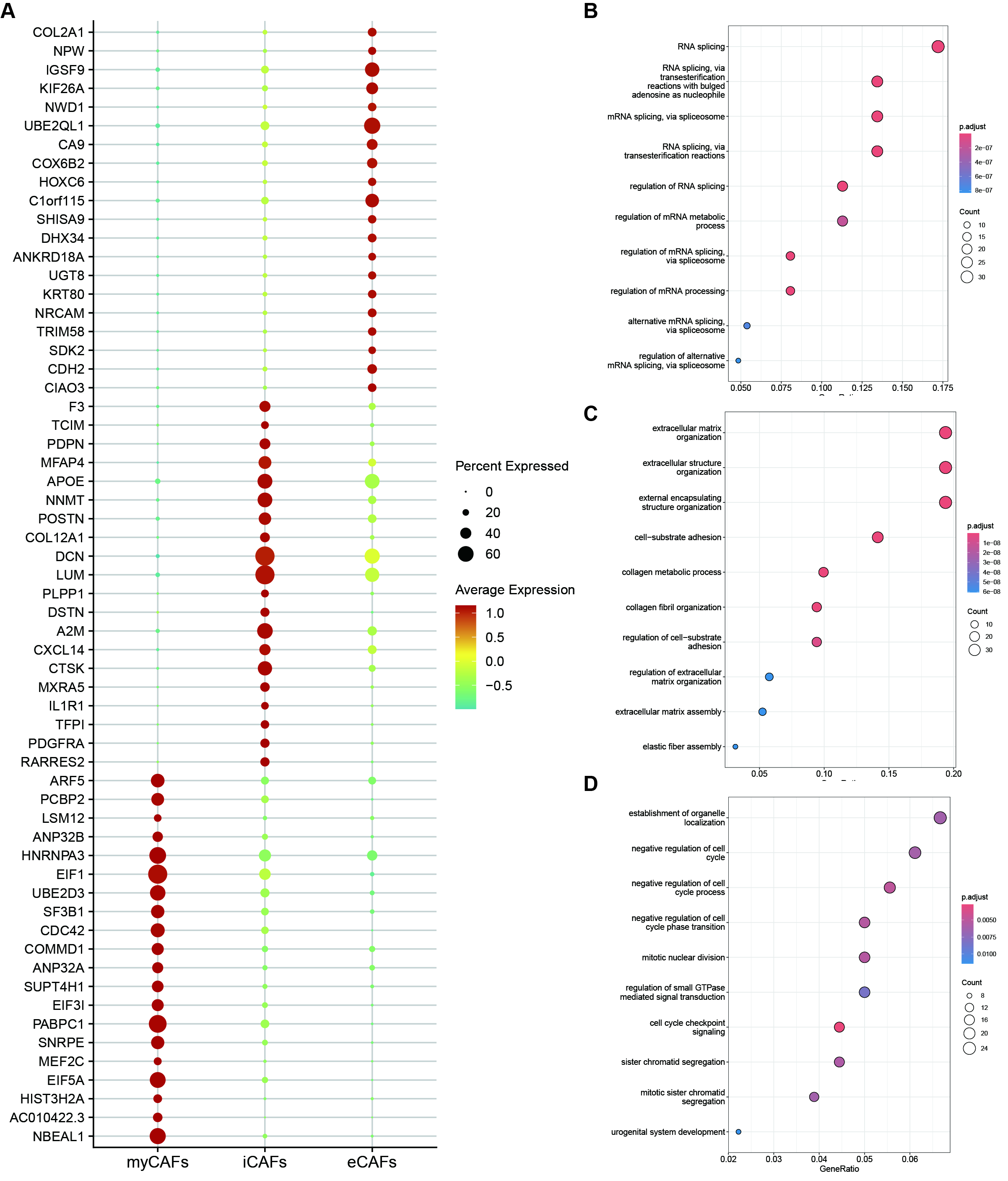

Supplement: Supplementary Figure 5 — Transcriptomic and functional differences among CAF subtypes. (A) Bubble plot showing the top 20 marker genes distinguishing myCAFs, iCAFs, and eCAFs. Dot size indicates the percentage of cells expressing the gene, and color represents average expression level. (B–D) GO enrichment analysis of differentially expressed genes in myCAFs (B), iCAFs (C), and eCAFs (D). Top enriched biological processes (BP) are displayed. Dot size represents the number of genes involved, and color denotes adjusted p-values. [file Image5.tif]

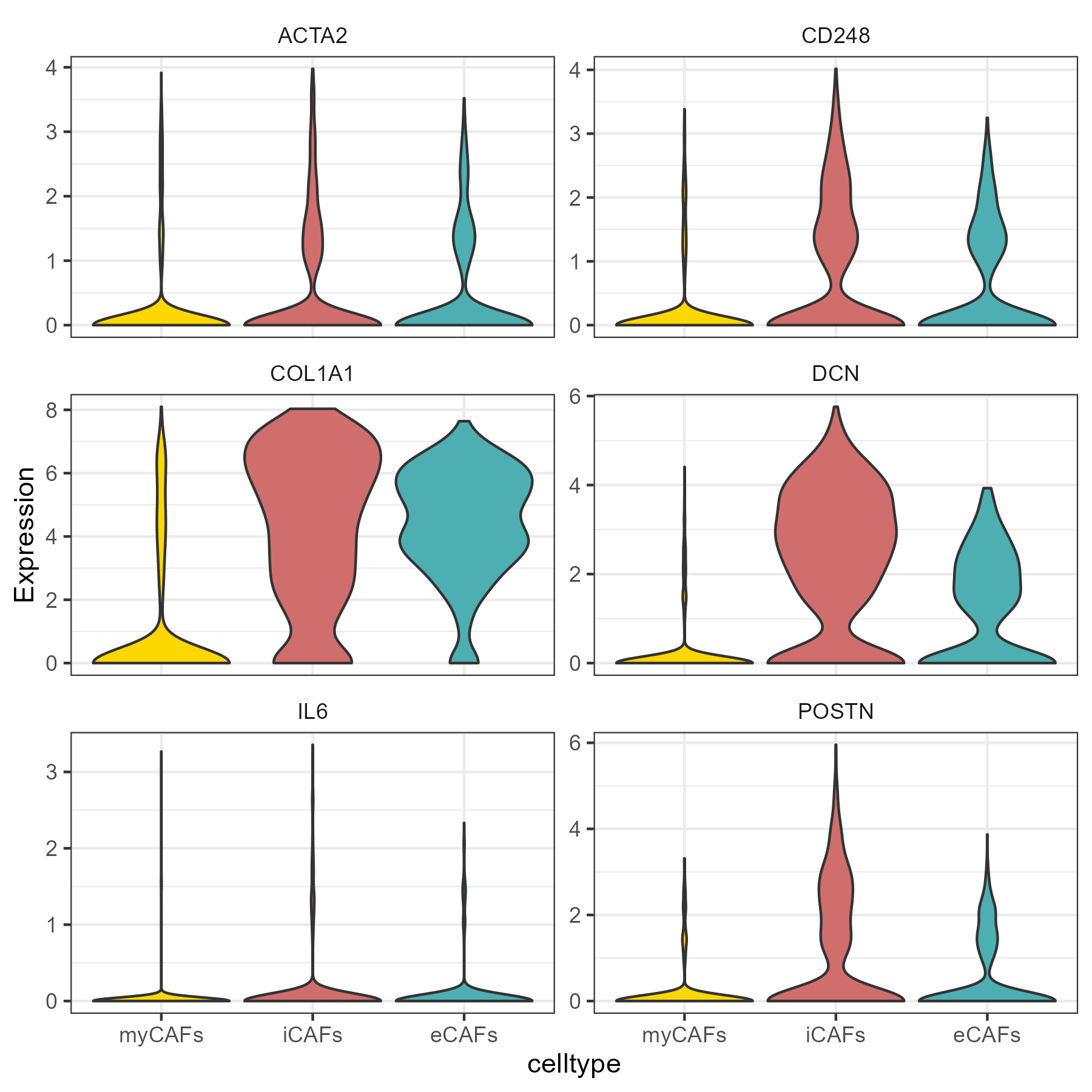

Supplement: Supplementary Figure 6 — Expression patterns of canonical CAF markers across fibroblast subtypes. Violin plots showing the expression patterns of six canonical CAF-associated markers (ACTA2, CD248, COL1A1, DCN, IL6, and POSTN) across the three CAF subtypes identified in SCCE. [file Image6.tif]
